# Supplementary material for: Follicular T cells are clonally and transcriptionally distinct in B cell-driven mouse autoimmune disease
Source: Nat Commun. 2021 Nov 18;12:6687. doi: 10.1038/s41467-021-27035-8 (PMC8602266; doi:10.1038/s41467-021-27035-8)
Supplement: Supplementary file 1 — Supplementary Information [file 41467_2021_27035_MOESM1_ESM.pdf]

Follicular T Cells are Clonally and Transcriptionally Distinct in B Cell-Driven Autoimmune Disease

Elliot H Akama-Garren, Theo van den Broek, Lea Simoni, Carlos Castrillon, Cees E. van der Poel, Michael C Carroll

Supplementary Information

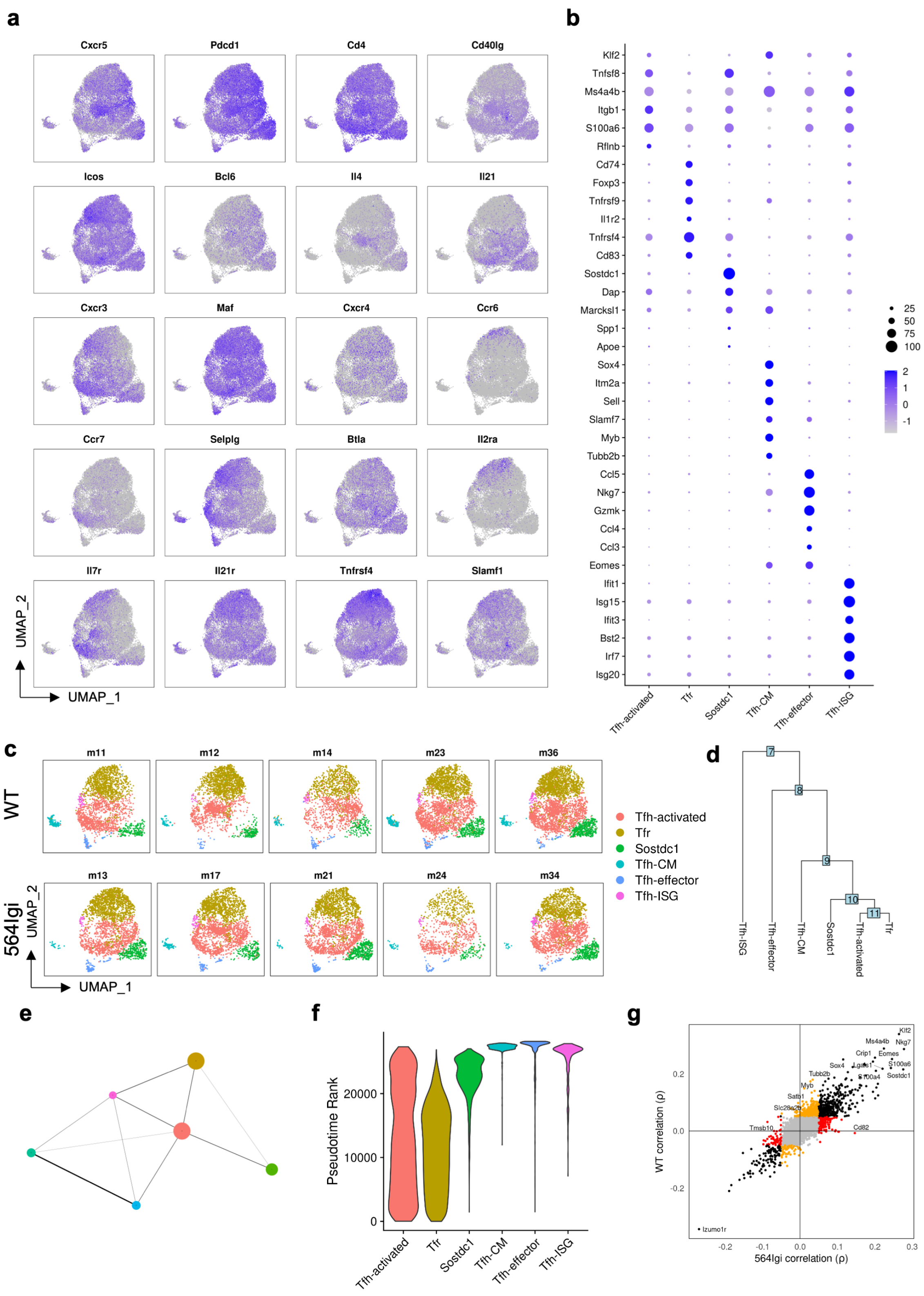

a
Cxcr5
Pdcd1
Cd4
Cd40lg
Icos
Bcl6
Il4
Il21
Cxcr3
Maf
Cxcr4
Ccr6
Ccr7
Selplg
Btla
Il2ra
Il7r
Il21r
Tnfrsf4
Slamf1
UMAP_2
UMAP_1
b
Klf2
Tnfsf8
Ms4a4b
Itgb1
S100a6
Rflnb
Cd74
Foxp3
Tnfrsf9
Il1r2
Tnfrsf4
Cd83
Sostdc1
Dap
Marcksl1
Spp1
Apoe
Sox4
Itm2a
Sell
Slamf7
Myb
Tubb2b
Ccl5
Nkg7
Gzmk
Ccl4
Ccl3
Eomes
Ifit1
Isg15
Ifit3
Bst2
Irf7
Isg20
25
50
75
100
2
1
0
-1
Tfh-activated
Tfr
Sostdc1
Tfh-CM
Tfh-effector
Tfh-ISG
c
WT
m11
m12
m14
m23
m36
564Igi
m13
m17
m21
m24
m34
UMAP_2
UMAP_1
Tfh-activated
Tfr
Sostdc1
Tfh-CM
Tfh-effector
Tfh-ISG
d
7
8
9
10
11
Tfh-ISG
Tfh-effector
Tfh-CM
Sostdc1
Tfh-activated
Tfr
e
f
Pseudotime Rank
20000
10000
0
Tfh-activated
Tfr
Sostdc1
Tfh-CM
Tfh-effector
Tfh-ISG
g
WT correlation (ρ)
564Igi correlation (ρ)
0.2
0.0
-0.2
-0.2
-0.1
0.0
0.1
0.2
0.3
Klf2
Ms4a4b
Nkg7
Crip1
Eomes
Sox4
Lgals1
S100a6
S100a4
Sostdc1
Tubb2b
Myb
Satb1
Slc28a2b
Tmsb10
Cd82
Izumo1r

Supplementary Fig. 1. Clustering is similar between autoimmune and non-autoimmune samples.

a, Gene expression of Tfh and Tfr defining genes projected onto UMAP of follicular T cells.

b, Dot plot of averaged log-normalized expression of top six differentially expressed genes (rows) for each cluster (columns). Size represents percentage of cells in cluster expressing gene.

c, UMAPs of follicular T cells split by individual samples of wild type (top) or autoimmune (bottom) chimeras, colored by cluster.

d, Hierarchical clustering of follicular T cell clusters.

e, Trajectory analysis computed using PAGA graph of paths between clusters.

f, Ranked pseudotime values of cells within clusters.

g, Scatter plot comparing correlation of gene expression with pseudotime values between WT or mixed 564Igi chimera follicular T cells.

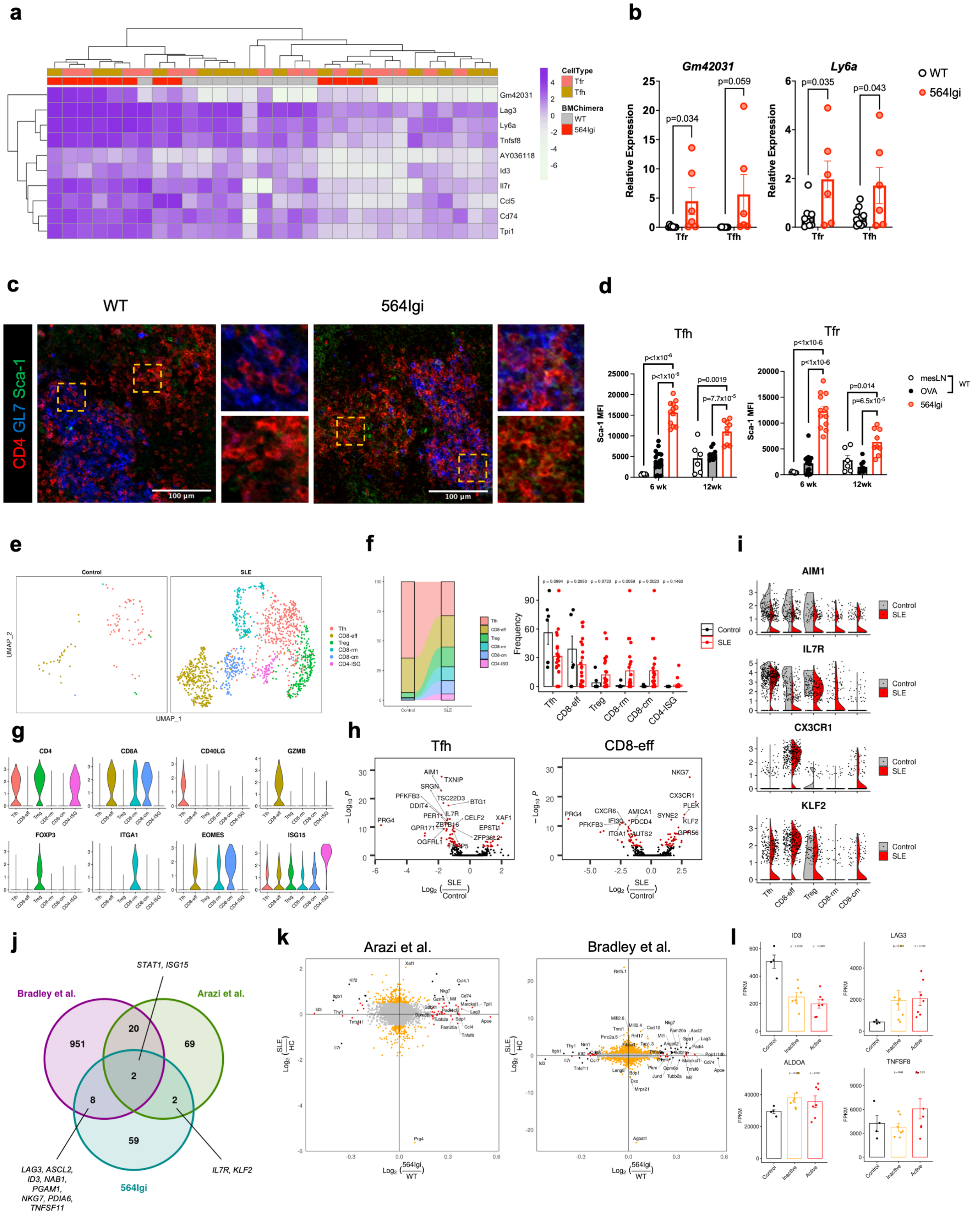
a
CellType
Tfr
Tfh
BMChimera
WT
564Igi
Gm42031
Lag3
Ly6a
Tnfsf8
AY036118
Id3
Il7r
Ccl5
Cd74
Tpi1
b
Gm42031
Ly6a
Relative Expression
p=0.034
p=0.059
p=0.035
p=0.043
Tfr
Tfh
WT
564Igi
c
WT
564Igi
CD4 GL7 Sca-1
100 µm
d
Tfh
Tfr
Sca-1 MFI
6 wk
12wk
mesLN
OVA
564Igi
WT
e
Control
SLE
UMAP_1
UMAP_2
Tfh
CD8-eff
Treg
CD8-rm
CD8-cm
CD4-ISG
f
Frequency
Control
SLE
g
CD4
CD8A
CD40LG
GZMB
FOXP3
ITGA1
EOMES
ISG15
h
Tfh
CD8-eff
i
AIM1
IL7R
CX3CR1
KLF2
j
Bradley et al.
Arazi et al.
564Igi
STAT1, ISG15
LAG3, ASCL2, ID3, NAB1, PGAM1, NKG7, PDIA6, TNFSF11
IL7R, KLF2
951
20
69
2
8
2
59
k
Arazi et al.
Bradley et al.
l
ID3
LAG3
ALDOA
TNFSF8
FPKM
Control
Inactive
Active

Supplementary Fig. 2. Follicular T cells are transcriptionally distinct in lupus patients.

a, Gene expression of candidate genes (rows) in Tfh ($CD3^+CD4^+CXCR\text{-}5^+PD\text{-}1^+GITR^-$) and Tfr ($CD3^+CD4^+CXCR\text{-}5^+PD\text{-}1^+GITR^+$) cells (columns) sorted from WT (grey, *n* = 9) or mixed 564Igi (red, *n*= 6) chimeras as measured by qRT-PCR. Unbiased hierarchical clustering of samples and genes shown. Gene expression is log-normalized relative to *Ywhaz*.

b, Expression of indicated genes in Tfh and Tfr cells sorted from WT (grey, *n* = 9) or mixed 564Igi (red, *n*= 6) chimeras as measured by qRT-PCR. P-value computed using two-tailed Student's *t*-test.

c, Confocal microscopy of spleens from WT (black, *n* = 9) or mixed 564Igi (red, *n* = 10) chimeras stained for CD4 (red), germinal centers (GL-7, blue), and Sca-1 (green). Insets(right) show higher magnification.

d, Flow cytometry quantification of Sca-1 expression in Tfh and Tfr cells from WT mesenteric lymph nodes (black circles, *n* = 7 for 6 weeks, *n* = 6 for 12 weeks), WT mice immunized with NP-OVA for indicated lengths (black, *n* = 16 for 6 weeks, *n* = 9 for 12 weeks), or 564Igi (red, *n* = 12 for 6 weeks, *n* = 9 for 12 weeks) mice at indicated ages. MFI, median fluorescence intensity. P-value computed using two-tailed Student's *t*-test.

e, UMAP visualization of T cells from renal biopsies of healthy control (left, *n* = 7) or systemic lupus erythematosus (SLE, right, *n* = 24) patients colored by unbiased cluster assignment. Raw scRNA-seq data obtained from Immport SDY997.

f, Stacked bar graph (left) and per sample average (right) of percent of T cells belonging to each cluster between healthy control (black) and SLE (red) patients.

g, Expression of cluster-defining genes within individual clusters.

h, Volcano plots of differentially expressed genes between control and SLE renal biopsy T cells within indicated clusters. Adjusted P-value <0.001 and $\log_2$FC >0.5 shown in red. Differential expression computed by MAST and adjusted for multiple comparison based on Bonferroni correction.

i, Expression of indicated genes within individual clusters amongst renal biopsy T cells split by control (grey) or SLE (red) samples.

j, Venn diagram comparing differentially expressed genes between $CD4^+$ PBMCs from healthy and SLE patients (Bradley et al.), between renal biopsy T cells from healthy and SLE patients (Arazi et al.), and between splenic follicular T cells from wild type and mixed 564Igi chimeras (564Igi). Mouse genes were converted to human orthologs where possible.

k, Scatter plot comparing log fold change of gene expression between mixed 564Igi and WT chimera follicular T cells with log fold change of gene expression between Tfh cells from SLE and healthy samples from renal biopsies (Arazi et al.) or $CD4^+$ PBMCs (Bradley et al.).

l, FPKM of indicated genes from RNA-sequencing of $CD4^+$ PBMCs from control (black, n = 4), inactivate (SLEDAI $\leq$6, orange, n = 7), and active (SLEDAI >6, red, n = 7) SLE patients.

Data are represented as mean ± SEM. Source data are provided as a Source Data file.

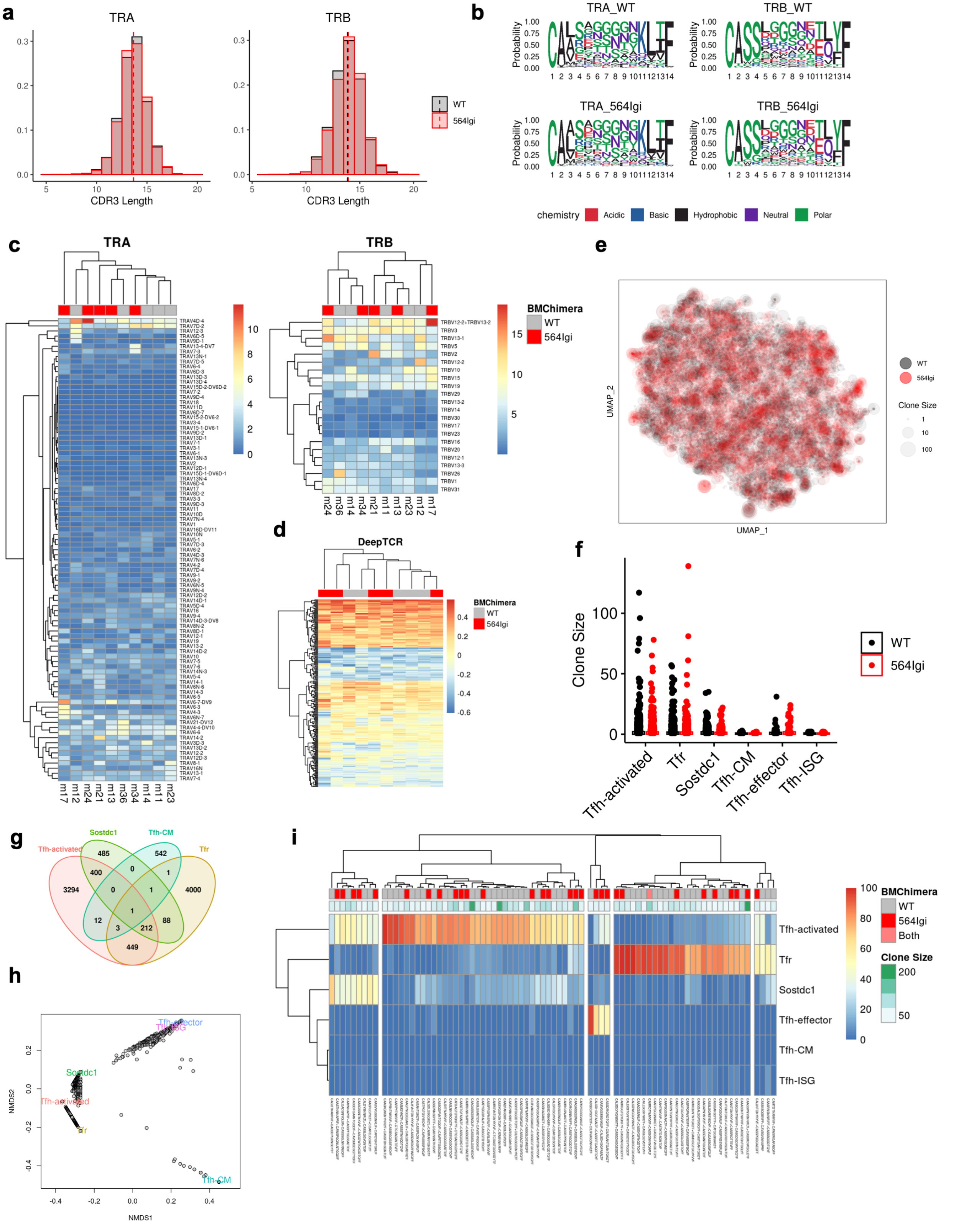
a
TRA
TRB
CDR3 Length
WT
564Igi
b
TRA_WT
TRB_WT
TRA_564Igi
TRB_564Igi
Probability
chemistry
Acidic
Basic
Hydrophobic
Neutral
Polar
c
TRA
TRB
BMChimera
WT
564Igi
d
DeepTCR
BMChimera
WT
564Igi
e
UMAP_2
UMAP_1
WT
564Igi
Clone Size
f
Clone Size
WT
564Igi
Tfh-activated
Tfr
Sostdc1
Tfh-CM
Tfh-effector
Tfh-ISG
g
Tfh-activated
Sostdc1
Tfh-CM
Tfr
h
NMDS1
NMDS2
Tfh-effector
Tfh-ISG
Sostdc1
Tfh-activated
Tfr
Tfh-CM
i
BMChimera
WT
564Igi
Both
Clone Size
Tfh-activated
Tfr
Sostdc1
Tfh-effector
Tfh-CM
Tfh-ISG

Supplementary Fig. 3. Clonotypes are infrequency shared between follicular T cell clusters.

a, Distribution of CDR3 length of TCRα (left) and TCRβ (right) chains of follicular T cells from WT (black) or mixed 564Igi (red) chimeras. Dashed vertical line indicates mean.

b, Motif analysis of 14 amino acid long CDR3s of TCRα (left) and TCRβ (right) chains of follicular T cells from WT (top) or mixed 564Igi (bottom) chimeras.

c, Hierarchical clustering of samples (columns) based on variable gene usage (rows) in TCRα (left) or TCRβ (right) chains. Row labels indicate variable gene name and column color indicates sample condition (grey, WT; red, 564Igi).

d, Hierarchical clustering of samples (columns) based on learned variable autoencoder-based feature space (rows) of TCR repertoires from WT (grey) or mixed 564Igi (red) chimeras.

e, UMAP visualization learned from TCR repertoire featurization performed on WT (black) and mixed 564Igi (red) chimeras. Individual clonotypes are depicted as circles and sized according to number of cells belonging to given clonotype. Points are colored according to condition.

f, Number of individual cells belonging to each clonotype within follicular T cell clusters identified by scRNA-seq amongst WT (black) or mixed 564Igi (red) chimeras.

g, Venn diagram comparing number of unique and shared clonotypes between follicular T cell clusters identified by scRNA-seq.

h, Ordinance plot of non-metric multidimensional scaling (NMDS) of clonotype distribution amongst individual samples. Text indicates sample name and color

indicates sample condition (grey, WT; red, 564Igi). Individual clonotypes are depicted as grey circles.

i, Hierarchical clustering of expanded clonotypes (>30 individual cells, columns) based on cluster distribution (rows). Column colors indicate sample condition of given clonotype (grey, WT; red, 564Igi; light red, both) and number of individual cells belonging to each clonotype. Columns are labeled with CDR3 sequences from TCR$\alpha\beta$ pairings.

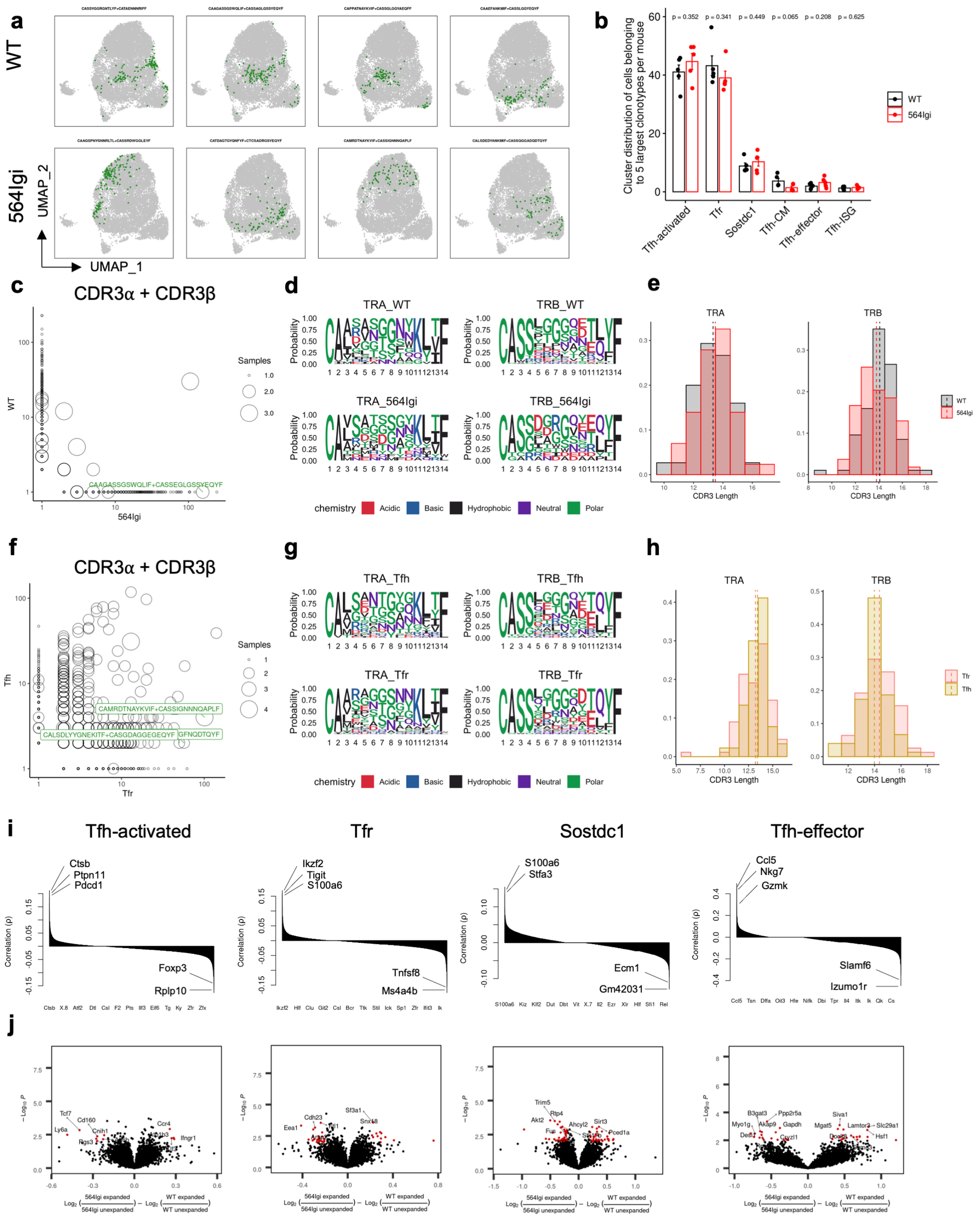
a
WT
564Igi
UMAP_2
UMAP_1
b
Cluster distribution of cells belonging to 5 largest clonotypes per mouse
p = 0.352
p = 0.341
p = 0.449
p = 0.065
p = 0.208
p = 0.625
WT
564Igi
Tfh-activated
Tfr
Sostdc1
Tfh-CM
Tfh-effector
Tfh-ISG
c
CDR3α + CDR3β
Samples
WT
564Igi
CAAGASSGSWQLIF+CASSEGLGSSYEQYF
d
TRA_WT
TRB_WT
TRA_564Igi
TRB_564Igi
Probability
chemistry
Acidic
Basic
Hydrophobic
Neutral
Polar
e
TRA
TRB
CDR3 Length
f
CDR3α + CDR3β
Samples
Tfh
Tfr
CAMRDTNAYKVIF+CASSIGNNNQAPLF
CALSDLYYGNEKITF+CASGDAGGEGEQYF
g
TRA_Tfh
TRB_Tfh
TRA_Tfr
TRB_Tfr
Probability
chemistry
Acidic
Basic
Hydrophobic
Neutral
Polar
h
TRA
TRB
CDR3 Length
Tfr
Tfh
i
Tfh-activated
Tfr
Sostdc1
Tfh-effector
Correlation (ρ)
Ctsb
Ptpn11
Pdcd1
Foxp3
Rplp10
Ikzf2
Tigit
S100a6
Tnfsf8
Ms4a4b
S100a6
Stfa3
Ecm1
Gm42031
Ccl5
Nkg7
Gzmk
Slamf6
Izumo1r
j
564Igi expanded
564Igi unexpanded
WT expanded
WT unexpanded

Supplementary Fig. 4. Clonal expansion amongst clusters is similar between autoimmune and non-autoimmune samples.

a, Mappings of four larges clonotypes in WT (top) or mixed 564Igi (bottom) chimeras onto UMAP visualization of transcriptomic data. CDR3 sequences of TCRαβ pairing of given clonotype indicated above each plot.

b, Percent of cells belonging to five largest clonotypes in each mouse assigned to each cluster in WT (top, n = 5) or mixed 564Igi (bottom, n = 5) chimeras. Data are represented as mean ± SEM. P-value computed using two-tailed Student's *t*-test.

c, Scatter plot comparing clone size between WT and mixed 564Igi chimeras with selected clonotypes labeled with CDR3 sequences from TCRαβ pairings. Clonotype size represents number of samples in which the given clonotype is observed.

d, Motif analysis of 14 amino acid long CDR3s of TCRα (left) and TCRβ (right) chains of cells belonging to preferentially expanded clonotypes indicated in Fig. 4b from WT (top) or mixed 564Igi (bottom) chimeras.

e, Distribution of CDR3 length of TCRα (left) and TCRβ (right) chains of cells belonging to preferentially expanded clonotypes indicated in Fig. 4b from WT (black) or mixed 564Igi (red) chimeras. Dashed vertical line indicates mean.

f, Scatter plot comparing clone size between Tfr and Tfh cells identified by scRNA-seq labeled with CDR3 sequences from TCRαβ pairings. Clonotype size represents number of samples in which the given clonotype is observed.

g, Motif analysis of 14 amino acid long CDR3s of TCRα (left) and TCRβ (right) chains of Tfh (top) or Tfr (bottom) cells identified by scRNA-seq.

h, Distribution of CDR3 length of TCRα (left) and TCRβ (right) chains of Tfh or Tfr cells. Dashed vertical line indicates mean.

i, Rank order plot of correlation coefficients of each gene with clone size across cells assigned to indicated cluster by scRNA-seq.

j, Volcano plots of differences between mixed 564Igi and WT chimeras in gene enrichment between expanded (>5 individual cells) and unexpanded (1 individual cell) clonotypes within indicated cluster. Adjusted P-value <0.01 and log_2FC >0.2 shown in red. Differential expression computed by MAST and adjusted for multiple comparison based on Bonferroni correction.

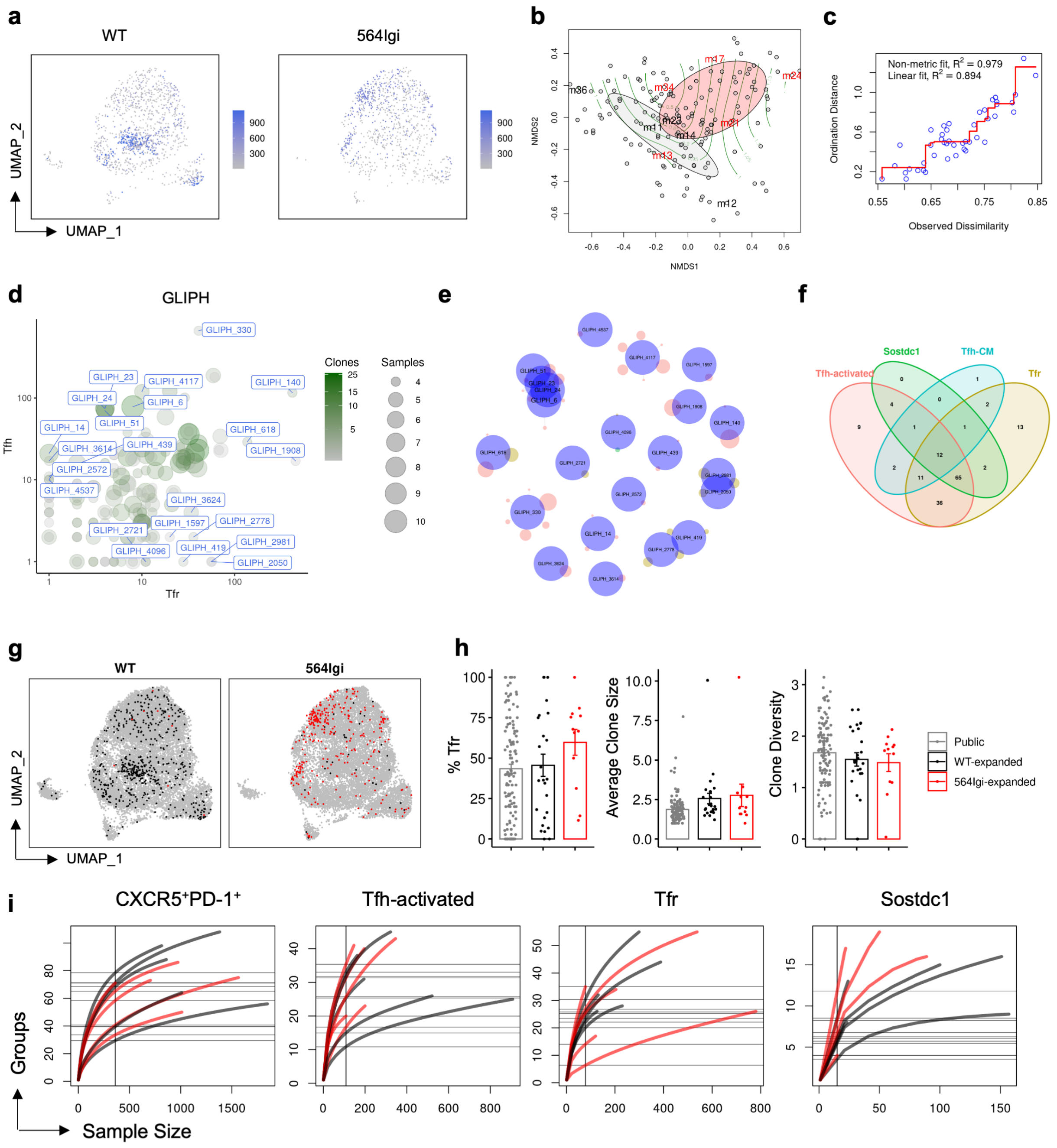

a
WT
564Igi
UMAP_2
UMAP_1
b
NMDS1
NMDS2
c
Non-metric fit, R^2 = 0.979
Linear fit, R^2 = 0.894
Ordination Distance
Observed Dissimilarity
d
GLIPH
Clones
Samples
Tfh
Tfr
e
f
Sostdc1
Tfh-CM
Tfh-activated
Tfr
g
WT
564Igi
UMAP_2
UMAP_1
h
% Tfr
Average Clone Size
Clone Diversity
Public
WT-expanded
564Igi-expanded
i
CXCR5+PD-1+
Tfh-activated
Tfr
Sostdc1
Groups
Sample Size

Supplementary Fig. 5. GLIPH analysis predicts frequent cross-reactivities between follicular T cell clusters.

a, Total size of specificity groups mapped onto UMAP visualization of transcriptomic data of individual follicular T cells from WT (left) or mixed 564Igi (right) mice.

b, Ordinance plot of non-metric multidimensional scaling (NMDS) of specificity group distribution amongst individual samples. Text indicates sample name and color indicates sample condition (grey, WT; red, 564Igi). Individual specificity groups are depicted as grey circles.

c, Shepard plot of ordination distances from non-metric multidimensional scaling (NMDS) of specificity group distribution amongst individual samples in (**b**).

d, Scatter plot comparing specificity group size between Tfr and Tfh cells. Specificity groups are colored according to the number of unique clonotypes that belong to a given specificity group and sized according to number of samples in which the given specificity group is observed. Select specificity groups are labeled with their arbitrary name in blue.

e, Unweighted network analysis of clonotype assignment to indicated specificity groups. Specificity groups are depicted as blue circles, clonotypes are colored according to most frequently assigned cluster and sized according to number of cells belonging to given clonotype.

f, Venn diagram comparing number of unique and shared specificity group between follicular T cell clusters identified by scRNA-seq.

g, Mappings of cells belonging to specificity groups preferentially expanded ($|\log_2 FC|>2.5$ and total size >10) in WT (black) or mixed 564Igi (red) chimeras onto

UMAP visualization of transcriptomic data from WT (left) or mixed 564Igi (right) chimeras.

h, Percentage of cells assigned to the Tfr cluster within each specificity group (left), geometric mean of clone sizes of clonotypes within each specificity group (middle), and Shannon diversity index of clonotype expansion within given specificity group (right) between specificity groups without a condition preference (public, grey, n = 121) or preferentially expanded in WT (black, n = 26) or mixed 564Igi (red, n = 12) chimeras. Data are represented as mean ± SEM.

i, Rarefaction curves of specificity group richness within follicular T cell clusters identified by scRNA-seq. Individual samples are depicted by lines and colored by sample condition (black, WT; red, 564Igi).

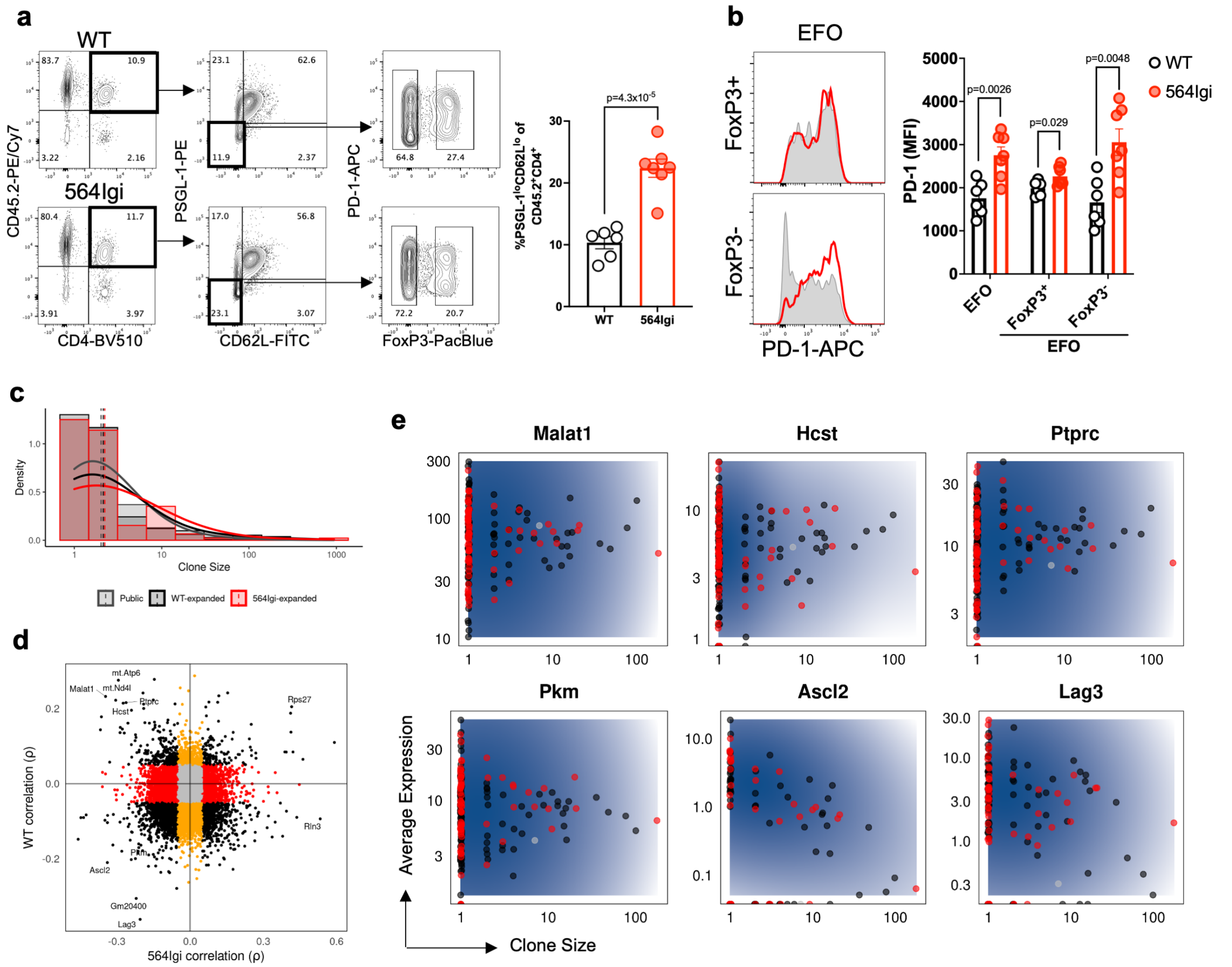

f

GLIPH_506

CDR3α	CDR3β	Clone Size	Sample	Condition
CAAGASSGSWQLIF	CASSAGLGSSYEQYF	226	m12	WT
CAAGASSGSWQLIF	CASSEGLGSSYEQYF	130	m24	564Igi
CAAGASSGSWQLIF	CASSEGLGSSYEQYF	130	m21	564Igi
CAAGASSGSWQLIF	CASSEGLGSSYEQYF	25	m24	564Igi
CAAGASSGSWQLIF	CASSEGLGSSYEQYF	25	m21	564Igi
CAALNQGGSAKLIF	CASSVGLGSSYEQYF	3	m11	WT
CAVRSYGNEKITF	CASSEGLGSSYEQYF	3	m21	564Igi
CAAGGANTGKLTF	CASSGGLGSSYEQYF	2	m13	564Igi
CAAGASSGSWQLIF	CASSEGLGSSYEQYF	1	m24	564Igi
CAAGASSGSWQLIF	CASSEGLGSSYEQYF	1	m21	564Igi

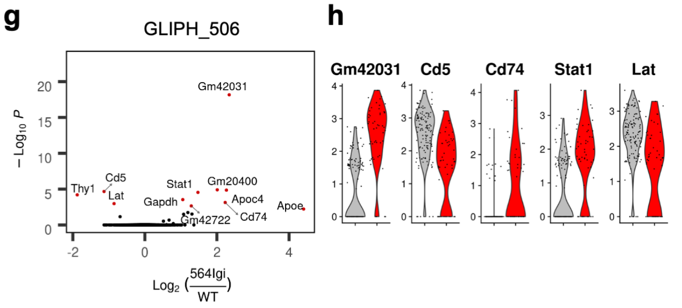

Supplementary Fig. 6. Autoimmune follicular T cells are transcriptionally distinct within a shared TCR specificity group.

a, Gating strategy (left) and quantification (right) to identify extrafollicular (EFO) $CD4^+$ T cells from WT (black, n = 6) or mixed 564Igi (red, n = 7) chimeras. Data are represented as mean ± SEM. P-value computed using two-tailed Student's *t*-test.

b, Flow cytometry histograms (left) and quantification (right) of PD-1 expression in EFO $CD4^+$ T cells from WT (black, n = 6) or mixed 564Igi (red, n = 7) chimeras. Data are represented as mean ± SEM. P-value computed using two-tailed Student's *t*-test.

c, Distribution of clone sizes amongst clonotypes belonging to specificity groups without a condition preference (public, grey) or preferentially expanded in WT (black) or mixed 564Igi (red) chimeras. Vertical lines indicate geometric means.

d, Scatter plot comparing correlation coefficients of each gene with clone size between WT and mixed 564Igi chimeras amongst cells belonging to specificity groups preferentially expanded in WT or mixed 564Igi chimeras. Correlation coefficients >0.05 are indicated in black (correlated in both conditions), red (correlated in mixed 564Igi chimera specificity groups only), or red (correlated in WT chimera specificity groups only).

e, Average expression of indicated gene amongst all cells belonging to individual clonotypes belonging to specificity groups preferentially expanded in WT or mixed 564Igi chimeras versus clone size. Clonotype color indicates sample condition (black, WT; red, 564Igi).

f, CDR3 sequences of clonotypes belonging to specificity group “GLIPH_506”. Conserved motif residues are highlighted, low contact probability residues indicated in red.

g, Volcano plot of differentially expressed genes between cells from mixed 564Igi versus WT chimera belonging to specificity group “GLIPH_506”. Adjusted P-value <0.01 and $\log_2$FC >0.2 shown in red. Differential expression computed by MAST and adjusted for multiple comparison based on Bonferroni correction.

h, Violin plot of expression of indicated genes in cells belonging to specificity group “GLIPH_506” in WT (grey) or mixed 564Igi (red) chimeras.

Source data are provided as a Source Data file.

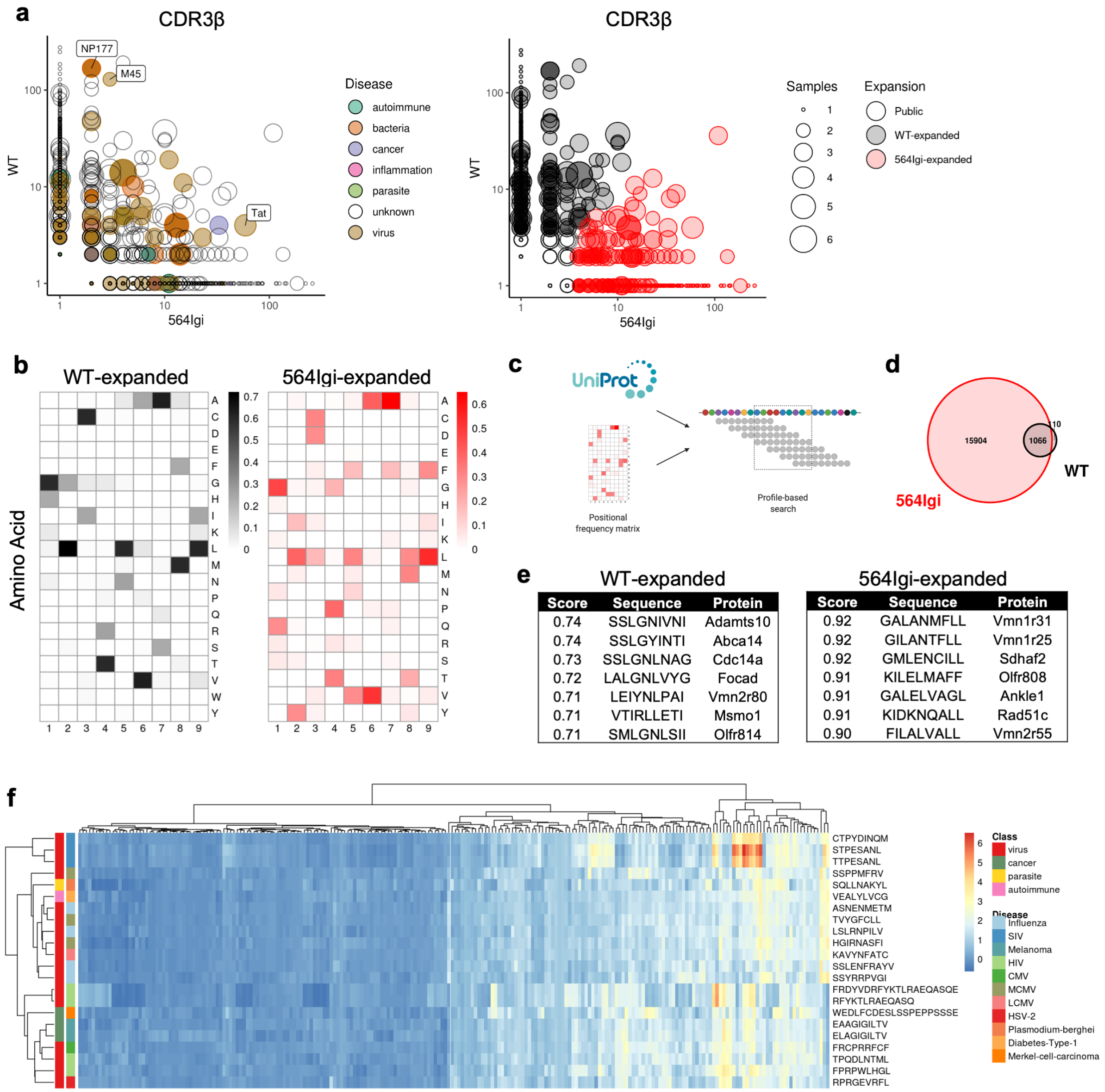
a
CDR3β
NP177
M45
Tat
WT
564Igi
Disease
autoimmune
bacteria
cancer
inflammation
parasite
unknown
virus
CDR3β
Samples
Expansion
Public
WT-expanded
564Igi-expanded
b
WT-expanded
564Igi-expanded
Amino Acid
c
UniProt
Positional frequency matrix
Profile-based search
d
15904
1066
110
564Igi
WT
e
WT-expanded
Score Sequence Protein
0.74 SSLGNIVNI Adamts10
0.74 SSLGYINTI Abca14
0.73 SSLGNLNAG Cdc14a
0.72 LALGNLVYG Focad
0.71 LEIYNLPAI Vmn2r80
0.71 VTIRLLETI Msmo1
0.71 SMLGNLSII Olfr814
564Igi-expanded
Score Sequence Protein
0.92 GALANMFLL Vmn1r31
0.92 GILANTFLL Vmn1r25
0.92 GMLENCILL Sdhaf2
0.91 KILELMAFF Olfr808
0.91 GALELVAGL Ankle1
0.91 KIDKNQALL Rad51c
0.90 FILALVALL Vmn2r55
f
CTPYDINQM
STPESANL
TTPESANL
SSPPMFRV
SQLLNAKYL
VEALYLVCG
ASNENMETM
TVYGFCLL
LSLRNPILV
HGIRNASFI
KAVYNFATC
SSLENFRAYV
SSYRRPVGI
FRDYVDRFYKTLRAEQASQE
RFYKTLRAEQASQ
WEDLFCDESLSSPEPPSSSE
EAAGIGILTV
ELAGIGILTV
FRCPRRFCF
TPQDLNTML
FPRPWLHGL
RPRGEVRFL
Class
virus
cancer
parasite
autoimmune
Disease
Influenza
SIV
Melanoma
HIV
CMV
MCMV
LCMV
HSV-2
Plasmodium-berghei
Diabetes-Type-1
Merkel-cell-carcinoma

Supplementary Fig. 7. Position-weighted search of protein databases based on known CDR3β specificities.

a, Scatter plot comparing CDR3β based clonotype size between WT and mixed 564Igi chimeras and colored according to disease category of predicted peptide from TCR database search of annotated CDR3β sequences (left) or preferential expansion (|log2FC|>3 and total size >10) in WT (black) or mixed 564Igi (red) chimeras (right). Size represents number of samples in which the given CDR3β sequence is observed. Labels represent antigens for selected CDR3β sequences present in our annotated TCR database.

b, Positional weighted matrix (PWM) of amino acid preference amongst nine amino acid long peptides corresponding to annotated CDR3β sequences from TCR databases that are identical to CDR3β clonotypes preferentially expanded in WT (left) or mixed 564Igi (right) chimeras identified by scRNA-seq. Peptides were weighted according to clone size multiplied by fold enrichment in given condition.

c, Schematic of PWM-based scoring of the mouse proteome to search for novel candidate peptides based on CDR3β annotation from (**b**).

d, Venn diagram of candidate antigens from the mouse proteome PWM-based search using annotated CDR3β clonotypes preferentially expanded in WT (black) or mixed 564Igi (red) chimeras.

e, Seven highest scoring peptides from the mouse proteome PWM-based search using annotated CDR3β clonotypes preferentially expanded in WT (left) or mixed 564Igi (right) chimeras.

f, Hierarchical clustering of annotated peptides (rows) based on learned feature space (columns) of CDR3β sequences from TCR databases using supervised training in Fig. 7G. Only peptides with AUC >0.9 are displayed. Row colors indicate disease class and disease information of given peptide.

Supplementary Table 1. Metadata and sequencing depth of scRNA-seq and scTCR-seq performed on wild type (WT) and mixed autoimmune (564Igi) bone marrow chimeras.

			scRNA-seq				scTCR-seq				
Mouse	Gender	Condition	Cells	Reads per cell	Genes per cell	Reads (million)	Cells	Reads per cell	Reads (million)	αβ Pair	Diversity
m14	M	WT	19148	15724	577	301.083152	6245	3384	21.13308	63.10%	539.7
m17	M	564Igi	6476	26083	1475	168.913508	4591	4832	22.183712	82.90%	118.5
m11	F	WT	5399	27779	1500	149.978821	4757	11962	56.903234	82.50%	350.36
m12	F	WT	4427	25186	1325	111.498422	3765	6591	24.815115	82.60%	77.78
m24	M	564Igi	3537	44891	980	158.779467	2642	12051	31.838742	77.50%	135.67
m13	F	564Igi	4756	21583	1551	102.648748	4084	8068	32.949712	86.80%	361.89
m23	M	WT	8269	21978	1228	181.736082	5638	7392	41.676096	83.30%	189.82
m21	F	564Igi	7352	23236	1082	170.831072	5105	9933	50.707965	78.80%	279.32
m34	F	564Igi	5027	30402	1368	152.830854	3155	21882	69.03771	67.90%	182.8
m36	M	WT	6840	26807	1361	183.35988	3914	16370	64.07218	66.40%	165.11

Supplementary Table 2. Primers for genotyping.

Primer name	Sequence
564Igi_H2_F	CACAGATTCTTAGTTTTTCAA
564Igi_H2_R	TGGAGCTATATCATCCTCTTT
564Igi_K_F	CCAGTGCAGATTTTCAGCTTC
564Igi_K_R	CAGCTTGGTCCCAGCACCGAA
mRPP30_F	TGACCCTATCAGAGGACTGC
mRPP30_R	CTCTGCAATTTGTGGACACG

Supplementary Table 3. Primers for qRT-PCR.

Primer name	Sequence
AY036118_F	CCAGCCAACGTAGAAAAGCC
AY036118_R	GCTCTTCCGTGTCTACGAGG
Ccl5_F	CTCACCATATGGCTCGGACACC
Ccl5_R	GCGGTTCCTTCGAGTGACAA
Cd74_F	ACTCCATGGATGGCGTGAAC
Cd74_R	TTCTTCCTGGCACTTGGTCAG
Gm42031_F	AGCTCGACTGCCAACAAGGAG
Gm42031_R	CAAAAAGCTGCTCCCATCGGT
Id3_F	TTTCCGAGAATGGGGTGTCG
Id3_R	ATCAGGGCAGCAGAGCTTTT
Il7r_F	CTGTCTTGCCAAGTGTCACC
Il7r_R	TTCAGACTATAGTTGTTCCAGAGTT
Lag3_F	GTAGCATCCATCTGCAGGGAC
Lag3_R	CAGATGCCGGGGTTACCTCA
Ly6a_F	CTCCCTCTTCAGGATGCCAG
Ly6a_R	GCACAGATAAAACCTAGCAGCTC
Tnfsf8_F	CCAGAAAAAGGACTCCACTCCA
Tnfsf8_R	ACTTGGAGGTAGGCCCATGA
Tpi1_F	GCCTGGGAGAACTCATCTGC
Tpi1_R	GCTTCTGTCTGGCAAAGTCG
Ywhaz_F	ACTTTTGGTACATTGTGGCTTCAA
Ywhaz_R	CCGCCAGGACAAACCAGTAT
