# Supplementary material for: Follicular T cells are clonally and transcriptionally distinct in B cell-driven mouse autoimmune disease
Source: Nat Commun. 2021 Nov 18;12:6687. doi: 10.1038/s41467-021-27035-8 (PMC8602266; doi:10.1038/s41467-021-27035-8)
Supplement: Supplementary file 3 — Description of Additional Supplementary Files [file 41467_2021_27035_MOESM3_ESM.pdf]

Description of Additional Supplementary Files

File Name: Supplementary Data 1
Description: **DEGs for follicular T cell clusters.** List of cluster-defining differentially expressed genes for each follicular T cell cluster defined by scRNA-seq.

File Name: Supplementary Data 2
Description: **DEGs between mixed autoimmune versus wild type chimera follicular T cells.** List of differentially expressed genes amongst follicular T cells from autoimmune (564Igi) versus wild type (WT) chimeras determined by scRNA-seq.

File Name: Supplementary Data 3
Description: **GLIPH2 specificity group assignments of clonotypes from scTCR-seq.** List of GLIPH2-based specificity group assignments of clonotypes identified by scTCR-seq of follicular T cells from autoimmune (564Igi) and wild type (WT) chimeras.

File Name: Supplementary Data 4
Description: **Reference database of known antigen specificities.** Compiled database of CDR3β sequences matched with known antigen specificities used for antigen specificity prediction.
