# Supplementary material for: Follicular T cells are clonally and transcriptionally distinct in B cell-driven mouse autoimmune disease
Source: Nat Commun. 2021 Nov 18;12:6687. doi: 10.1038/s41467-021-27035-8 (PMC8602266; doi:10.1038/s41467-021-27035-8)
Supplement: Supplementary file 8 — Reporting Summary [file 41467_2021_27035_MOESM8_ESM.pdf]

nature research

Corresponding author(s): Michael C. Carroll

Last updated by author(s): Jan 28, 2021

Reporting Summary

Nature Research wishes to improve the reproducibility of the work that we publish. This form provides structure for consistency and transparency in reporting. For further information on Nature Research policies, see our Editorial Policies and the Editorial Policy Checklist.

Statistics

For all statistical analyses, confirm that the following items are present in the figure legend, table legend, main text, or Methods section.

n/a	Confirmed	
☐	☒	The exact sample size (*n*) for each experimental group/condition, given as a discrete number and unit of measurement
☐	☒	A statement on whether measurements were taken from distinct samples or whether the same sample was measured repeatedly
☐	☒	The statistical test(s) used AND whether they are one- or two-sided<br>*Only common tests should be described solely by name; describe more complex techniques in the Methods section.*
☐	☒	A description of all covariates tested
☐	☒	A description of any assumptions or corrections, such as tests of normality and adjustment for multiple comparisons
☐	☒	A full description of the statistical parameters including central tendency (e.g. means) or other basic estimates (e.g. regression coefficient) AND variation (e.g. standard deviation) or associated estimates of uncertainty (e.g. confidence intervals)
☐	☒	For null hypothesis testing, the test statistic (e.g. *F*, *t*, *r*) with confidence intervals, effect sizes, degrees of freedom and *P* value noted<br>*Give P values as exact values whenever suitable.*
☒	☐	For Bayesian analysis, information on the choice of priors and Markov chain Monte Carlo settings
☒	☐	For hierarchical and complex designs, identification of the appropriate level for tests and full reporting of outcomes
☒	☐	Estimates of effect sizes (e.g. Cohen's *d*, Pearson's *r*), indicating how they were calculated

Our web collection on statistics for biologists contains articles on many of the points above.

Software and code

Policy information about availability of computer code

Data collection	Confocal images were acquired using Fluoview v4.2b software (Olympus).<br>Flow cytometry data was acquired using BD FACSDiva v9.0 software (BD Bioscience).<br>Real-time PCR data was acquired using QuantaSoft v1.7 software (BioRad).
Data analysis	All analysis were described in the relevant section of Methods.<br>Confocal images were analyzed using Fiji v2.1.0 software (ImageJ).<br>Flow cytometry data was analyzed using FlowJo v10 software (Treestar).<br>Single-cell RNAseq and TCRseq data was analyzed using CellRanger v4.0.0, GLIPH2, the R packages Seurat v3.1.4, vegan v2.5-6, qgraph v1.6.5, immunarch v0.5.5, ggseqlogo v0.1, mgsigdbr v7.0.1, clusterProfiler v.3.14.3, biomaRt v2.24.0, MAST v1.12.0 and the python packages scanpy v.1.5.1, anndata2ri v1.0.2, and DeepTCR v1.4.15.<br>Bulk RNAseq data was analyzed using the R package DEseq2 v1.26.0.<br>Data visualization was done using ggpubr v0.2.5.999, Prism v9.2.0 (GraphPad), vennDiagram v 1.6.20, ggplot2 v3.3.0, pheatmap v 1.0.12, ggseqlogo v0.1, and EnhancedVolcano v1.4.0.

For manuscripts utilizing custom algorithms or software that are central to the research but not yet described in published literature, software must be made available to editors and reviewers. We strongly encourage code deposition in a community repository (e.g. GitHub). See the Nature Research guidelines for submitting code & software for further information.

Data

Policy information about availability of data

All manuscripts must include a data availability statement. This statement should provide the following information, where applicable:

- Accession codes, unique identifiers, or web links for publicly available datasets
- A list of figures that have associated raw data
- A description of any restrictions on data availability

Single cell RNAseq and TCRseq data is available at accession code GSE157649.
The following publicly available datasets were used: Immport SDY997, Bradley et al. PLOS One 2015 (https://doi.org/10.1371/journal.pone.0141171.s003), VDJdb (https://vdjdb.cdr3.net/), PIRD (https://db.cngb.org/pird/), McPAS-TCR (http://friedmanlab.weizmann.ac.il/McPAS-TCR/), UniProt UP000000589, and GRCm38 reference genome (mm10).

Field-specific reporting

Please select the one below that is the best fit for your research. If you are not sure, read the appropriate sections before making your selection.

☒ Life sciences ☐ Behavioural & social sciences ☐ Ecological, evolutionary & environmental sciences

For a reference copy of the document with all sections, see nature.com/documents/nr-reporting-summary-flat.pdf

Life sciences study design

All studies must disclose on these points even when the disclosure is negative.

Sample size	No sample size calculation were performed. Pilot studies and previous characterization of the mixed bone marrow chimera model (Degn et al. Cell 2017) were used for estimation of the sample size required.
Data exclusions	No data were excluded from analyses.
Replication	All experiments were reproduced to reliably support conclusions stated int he manuscript. Each experiment was repeated in multiple animals and at least three times.
Randomization	Mice were randomly allocated into bone marrow chimera cohorts.
Blinding	For in vivo experiments the investigators were not blinded to group allocation as they performed both the experiment and analysis, blinding was not possible. Sequencing results were analyzed computationally and objectively, minimizing potential bias from lack of blinding.

Reporting for specific materials, systems and methods

We require information from authors about some types of materials, experimental systems and methods used in many studies. Here, indicate whether each material, system or method listed is relevant to your study. If you are not sure if a list item applies to your research, read the appropriate section before selecting a response.

Materials & experimental systems

n/a	Involved in the study	
☐	☒	Antibodies
☒	☐	Eukaryotic cell lines
☒	☐	Palaeontology and archaeology
☐	☒	Animals and other organisms
☒	☐	Human research participants
☒	☐	Clinical data
☒	☐	Dual use research of concern

Methods

n/a	Involved in the study	
☒	☐	ChIP-seq
☐	☒	Flow cytometry
☒	☐	MRI-based neuroimaging

Antibodies

Antibodies used	Biolegend: anti-CD44 (IM7) #103007, CD62L (MEL-14) #104405, CD45.1 (A20) #110721, CD45.2 (104) #109829, CXCR5 (L138D7) #145510, PD-1 (RMP1-30) #109112, Sca-1 (D7) #109105, GL7 (GL7) #144606, CD3 (500A2) #152311, GITR (DTA-1) #126318, and CD4 (GK1.5) #100427.<br><br>BD Biosciences: anti-PSGL-1 (2PH1) #555306<br><br>ThermoFisher: anti-FoxP3 (FJK-16s) #48-5773-82

Validation	The antibodies used in this study were validated by the manufacturer.

Animals and other organisms

Policy information about studies involving animals; ARRIVE guidelines recommended for reporting animal research

Laboratory animals	C57BL/6J and B6.SJL (CD45.1) mice were obtained from Jackson Laboratories. 564Igi mice were originally provided by Theresa Imanishi-Kari (Tufts University) and were maintained in-house. Males and females were used at adult (8-12 week) stages. All mice were fed and watered ad libitum with consistent access to food and water, and all mice were housed in a facility kept at ambient temperature and humidity with 12 hr light/12 hr dark cycles.
Wild animals	No wild animals were used in this study.
Field-collected samples	No field samples were collected in the study.
Ethics oversight	All animal experiments were conducted in accordance with the guidelines of the Laboratory Animal Center of National Institutes of Health. The Institutional Animal Care and Use Committee of Harvard Medical School approved all animal protocols (IS111).

Note that full information on the approval of the study protocol must also be provided in the manuscript.

Flow Cytometry

Plots

Confirm that:

☒ The axis labels state the marker and fluorochrome used (e.g. CD4-FITC).

☒ The axis scales are clearly visible. Include numbers along axes only for bottom left plot of group (a 'group' is an analysis of identical markers).

☒ All plots are contour plots with outliers or pseudocolor plots.

☒ A numerical value for number of cells or percentage (with statistics) is provided.

Methodology

Sample preparation	Spleens and lymph nodes were harvested into ice cold FACS buffer (PBS with 0.5% heat inactivated FBS and 0.05% sodium azide) and mechanically digested through a 70 um cell strainer (Corning). Spleens were incubated in RBC lysis buffer (155 mM NH4Cl, 12 mM NaHCO3, 0.1 mM EDTA) for 3 min at room temperature and washed with FACS buffer. Cells were counted and 1 x 10^6 cells/well were added to round-bottom 96 well plates and incubated with 50 uL of staining mix (appropriate antibodies and viability dye in FACS buffer) for 30 min on ice. Plates were washed with FACS buffer and for two-step staining procedures incubated with 50 uL of secondary staining mix (appropriate streptavidin antibody in FACS buffer) for 15 min on ice. For intracellular staining, cells were fixed with Fixation/Permeabilization Buffer (eBioscience) for 30 min at room temperature, washed with Permeabilization Buffer (eBioscience), and incubated with 50 uL of intracellular staining mix (appropriate intracellular antibody in Permeabilization Buffer) for 30 min at room temperature. Cells were washed with a final wash of FACS buffer, resuspended in 150 uL FACS buffer
Instrument	Analysis was performed using 3-8 fluorophore flow cytometry on a FACSCanto II (BD Biosciences) with 488, 405, and 640 nm lasers. Sorting was performed using a FACSARIA II Special Order system (BD Biosciences) with 355, 405, 488, 640, and 592 nm lasers.
Software	Flow cytometry data was collected using BD FACSDiva software (BD Bioscience) and analyzed using FlowJo software.
Cell population abundance	Post-sort sample purity was examined by flow cytometry, and confirmed to be at least 95% of desired population.
Gating strategy	Debris was removed using FSC/SSC and doublets were excluded SSC-H/SSC-W and FSC-H/FSC-W. Viability dye was used. Follicular T cells were gated on CD4+CXCR-5+PD-1+.

☒ Tick this box to confirm that a figure exemplifying the gating strategy is provided in the Supplementary Information.
